# Nutrient quality of vertebrate dung as a diet for dung beetles

**DOI:** 10.1038/s41598-017-12265-y

**Published:** 2017-09-22

**Authors:** Kevin Frank, Adrian Brückner, Andrea Hilpert, Michael Heethoff, Nico Blüthgen

**Affiliations:** Ecological Networks, Department of Biology, Technische Universität Darmstadt, Schnittspahnstraße 3, 64287 Darmstadt, Germany

## Abstract

At the basis of a trophic web, coprophagous animals like dung beetles (Scarabaeoidea) utilize resources that may have advantages (easy gain and handling) as well as drawbacks (formerly processed food). Several studies have characterized the nutrients, e.g. C/N ratios and organic matter content, for specific types of dung. However, a comparative approach across dung types and feeding guilds of dung producers, and relationships between dung nutrients and preferences by coprophages, have been missing. Hence, we analyzed water content, C/N ratio, amino acid, neutral lipid fatty acid, free fatty acid and sterol composition and concentrations in dung from 23 vertebrates, including carnivore, omnivore and herbivore species. Our analyses revealed significant differences among the three vertebrate feeding guilds for most nutritional parameters. Although formerly processed, dung grants sufficient amounts of essential nutrients for insects. We tested whether nutrients can explain the dung beetles’ preferences in a field experiment, using 12 representative dung types in baits that were installed in 27 forests and 27 grasslands. Although consistent preferences for specific dung types were pronounced, the nutritional composition did not predict the variation in attractiveness of these dung diets, suggesting a primary role of dung volatiles irrespective of food quality.

## Introduction

Heterotrophic organisms have to consume food to generate energy, grow and maintain metabolism^1^, thus various strategies for detection, foraging and processing to exploit a wide range of diets have evolved^2^. Moreover the patchy distribution of manifold resources and its constant dynamics selected for differences in feeding behaviours; ranging from opportunistic to highly specialized feeding^3^. Some animals mainly use the metabolic trash of others (dung) to fulfil their energy requirements. Such coprophages (=“dung eaters”) substantially contribute to nutrient and energy flows in ecosystems^4,5^, since “in nature, nothing is wasted – not even waste”^6^.

One of the most common invertebrate coprophages are dung beetles (Scarabaeoidea); a cosmopolitan superfamily of insects which evolved a detritivorous life-style several million years ago^7–10^. Although the ancient detritivorous feeding behaviour (using all organic material, including dung, litter, humus and carcasses) still exists in many families of Scarabaeoidea^11,12^, the increased occurrence of megafauna during the lower Jurassic has provided a new exploitable resource in large quantities, facilitating the evolution of dung beetles towards coprophagy^13^. Dung beetles are often generalistic in their use of different types of faeces, although certain dung types are clearly more frequently utilized than others^14^. Such preferences may be influenced and modified by the “host” animals’ diet (carnivore, herbivore, and omnivore) which affects the nutrients, volatile organic compounds or odour intensities in their faeces^11,14–18^. Dung nutrients are of particular importance in dung beetle development, e.g. for their body size or the length of the male’s horns^19,20^. Dung itself consists of metabolic waste products and undigested remains of the original food. However, also other food sources like humus, fungi and carrion are used by dung beetles^7^, thus questioning dung as the only trophic resource for dung beetles which can supplement them with all mandatory nutrients. Furthermore, endosymbiotic bacteria associated with dung beetles may facilitate the digestion of dung and could foster a well-balanced nutritional supplementation^21–23^ as regularly found in others insects^24–26^. Several studies analyzed C/N ratios, organic matter contents, amino acids as well as further components (recently reviewed by Holter^27^) and addressed the high variability of composition and nutritional values among dung types and feeding guilds^14^. However, a comparative approach analysing the nutrients, dung type preferences and nutrient-preference-relationships in a broad variety of dung types from different species has not been conducted so far.

Hence, we address the following questions; i) does the nutritional composition differ among the dung from different feeding guilds of vertebrates; ii) can this dung potentially supply all essential macronutrients for insects like dung beetles; iii) does the dung differ in its attractiveness to dung beetles, and if; iv) do preferences correspond to nutritional composition? We analyzed C/N ratios, amino and fatty acids, sterol and water contents of dung from 23 vertebrate species (7 carnivores, 6 omnivores and 10 herbivores) using gas-chromatography (GC), gas chromatography/mass spectrometry (GC/MS) and ion-exchange chromatography (IEC). Furthermore, we used 12 (out of the 23) dung types for a field experiment to compare the attractiveness of dung from different feeding guilds to dung beetles. We show that i) the nutritional composition of dung differed among feeding guilds, although ii) almost all essential macronutrients were found in all samples; iii) dung beetles showed significant dung type preferences; but iv) preferences did not correspond to nutritional parameters.

## Methods

### Dung sampling and processing

We used 23 different dung types of carnivorous (otter, lynx, mink, raven, snowy owl, wolf, wild cat), omnivorous (chicken, wild boar, brown bear, fox, gerbil, raccoon) and herbivorous (cow, donkey, deer, elephant, elk, goat, horse, rabbit, sheep, wisent) species, which we collected at two organic farms, wildlife parks/zoos and private stocks around Darmstadt (detailed information on origin and diet of each species are provided in the Supplementary Information; Table S1 and Supplementary Methods S1). To better represent each dung type, most dung samples originated from several individuals, and individual droppings were mixed. We sampled dung at different times and checked for intraspecific variation between samples in all nutrients. As expected, variation among measurements was much smaller than across dung types: the coefficient of variation of amino acids, fatty acids and sterols, C/N ratios and water content was 3.4–7.6-fold higher across dung types than within each dung type (Supplementary Table S2). Moreover, cow dung from 11 different farms clustered together in a discriminant analysis of principal components (Supplementary Figure S1; for details see statistical analysis). We thus disregard the intraspecific variation in the main results.

Animals involved in this study had not faced any veterinarian treatment for several weeks before dung collection. After collecting fresh samples (i.e. droppings from the collection day) in a sufficient amount, the dung was filled in a tea bag (for dung baits used in the field), subsequently transferred into a freezer bag, sealed and labelled. A part of the sampled dung was further processed for chemical analysis (see below). All samples were stored in a freezer at −20 °C until use.

### Water content and dry weight

Water content of each dung was determined with a microbalance (Mettler Toledo, XS3DU, Columbus OH, USA; readability 0.1 μg and 1 μg repeatability) two times with three replicates (20 mg, 50 mg and 100 mg) per dung; (a) the initial fresh mass of the dung prior to drying and (b) its dry mass after the dung has been dried until weight constancy in an oven at 60 °C for at least 4 days. For the determination of the dry weight of the bird faeces (raven and snowy owl), which were partly absorbed in sand, the actual dry organic matter was determined by fully annealing the dung samples with a Bunsen burner and back-weighing of the ignition residues. Linear regression formulas for the calculation of each dung’s dry weight are given in the Supplementary Information (List S1).

### Lipid and sterol analyses

Total neutral lipids (hereafter, neutral lipid fatty acids = NLFAs) were extracted from the fresh dung samples (40–50 mg of fresh weight) using 1 ml of a chloroform:methanol-mixture, 2:1 (V/V)^28^ over a period of 24 h. Afterwards two replicate extracts were purified and separated^29^,^30^ to fractionate neutral lipid fatty acids (NLFAs) and free fatty acids (FFAs), respectively (for detailed procedure see Supplementary Method S1). After the samples had been fractionated by column chromatography, they were finally measured with a QP2010 Ultra GC/MS (Shimadzu, Duisburg, Germany). The gas chromatograph (GC) was equipped with a ZB-5MS fused silica capillary column (30 m × 0.25 mm ID, df = 0.25 μm) from Phenomenex (Aschaffenburg, Germany). 1 μl sample aliquots were injected by using an AOC-20i autosampler-system (Shimadzu, Duisburg, Germany) into a PTV-split/splitless-injector (Optic 4, ATAS GL, Eindhoven, Netherlands), which operated in splitless-mode. Injection-temperature was programmed from initial 70 °C up to 300 °C and then an isothermal hold for 59 minutes. Hydrogen was used as carrier-gas with a constant flow rate of 1.5 ml/min. The temperature of the GC oven was raised from initial 60 °C for 1 min, to 150 °C with a heating-rate of 15 °C/min, to 260 °C with a heating-rate of 3 °C/min, to 320 °C with a heating-rate of 10 °C/min and then an isothermal hold at 320 °C for 10 min. Electron ionization mass spectra were recorded at 70 eV from *m/z* 40 to 650. The ion source of the mass spectrometer and the transfer line were kept at 250 °C. FAMEs were identified based on their retention indices^31^ and *m/z* fragmentation patters as well as by comparison with the FAME and BAME analytical standards (Sigma-Aldrich, St. Lois, USA). The configurations of the double bonds were not specifically determined. The amount of fatty acids (i.e. NLFAs and FFAs) [µg] was standardized using the dry weight [mg] calculated form the initial fresh weight of the sample.

Sterols were quantified based on the peak area of detected compounds relative to the constant amount of the internal standard (220 ng/µl nonadecanoic acid) expressed in [%] of this standard, because we did not determine the response factor of the sterols to the internal standard. Cholesterol was the only sterol that was identified based on its *m/z* fragmentation [as cholesteryl methyl ether: 400 (M^+^, 60); 385 (24); 368 (100); 353 (59), 329 (31), 301 (25), 275 (37), 213 (26), 145 (42), 107 (50), 81 (46), 69 (27), 55 (41)], for the other sterols we just checked for correct substance class assignment (as sterols) based on their mass spectra. The amounts of sterols [% Std.] and cholesterol [% Std.] were standardized using the dry weight [mg] calculated form the initial fresh weight of the sample. The cholesterol/sterol ratio [%] was calculated based on both values.

### Amino acid analysis

For analysis of the amino acids (free amino acids and protein-bounded), 5 mg (±0.1 mg) dried dung was diluted in 200 μL of hydrochloric acid (6 mol/l) and boiled for four hours at 100 °C, processed (for detailed procedure see Supplementary Method S1) and finally measured (﻿as described in^32^) with an ion exchange chromatograph with ninhydrin post-column derivatization (Biochrom 20+, Amino Acid Analyzer, Cambridge, UK). A standard amino acid mixture (Laborservice Onken GmbH, Gründau, Germany) was used as external standard. The amount of total amino acids [µg] was standardized using the dry weight [mg] of the initial sample. Note that this acidic chemical extraction decays asparagine, glutamine and tryptophan.

### C/N analysis

Dried dung samples were mixed with hydrochloric acid (HCl; approx. 0.05 mol/l) to remove the inorganic carbonate as CO_2_. Samples were subsequently dried again and weighed into tin capsules (6 ± 1 mg dry weight). Total organic carbon and nitrogen contents were measured by an elemental analyser (EA 1108 Elemental Analyser, Carlo Erba, Milan, Italy). Acetanilide (Merck, Darmstadt, Germany) was used for internal calibration.

### Field sampling and study site

For dung beetle field samplings we used pitfall traps equipped with the dung baits of 12 different representative subsamples (i.e. dung available in sufficient amounts) collected from mammal species, namely: wolf, lynx, fox, brown bear, wild boar, cow, horse, sheep, deer, elephant, elk and wisent (2 carnivores, 3 omnivores and 7 herbivores). Six dung types commonly occur in the sampled regions (game in forests and grasslands: fox, deer, wild boar, livestock on pastures: cow, horse, sheep). However, previous studies showed there is no effect of conditioning for dung beetles, when raised on a certain dung type^33^. The traps were set up randomized on a transect, in a total of 54 experimental sites (27 in forests, 27 in grasslands) in three regions of Germany within the Biodiversity Exploratories’ framework (see Supplementary Method S1). All dung baits have been taken out of the freezer approx. one hour before placing them in the field, which ensures an equal defrosting time and temperature for the samples (mean temperature during field sampling: 19.5 °C). All field samplings were performed between 29th June and 17th July 2015. Pitfall traps were collected after 48 hours, trapped beetles were labelled (date, site-ID, dung type) and stored in a freezer at −20 °C. Dung beetles were identified to species level using the keys of Freude, *et al*.^34^, Bunalski^35^ and Rössner^36^, and confirmed by taxonomic experts (see Acknowledgements). All methods were carried out in accordance with relevant guidelines and regulations, and sampling of dung and beetles was approved by local authorities, see Acknowledgements.

### Data processing and statistical analyses

Preferences of dung beetles across different dung were analyzed on three different levels: i) the number of dung beetles collected per plot (dependent variable) on a certain dung type (independent variable) was analysed using Friedman test with plot ID as blocking factor. ii) The number of dung beetles collected on each plot/number of traps (dependent variable) on dung of the three different feeding guilds (independent variable) was analyzed using Friedman test with plot ID as blocking factor, as well. iii) The proportional abundance [%] of different beetle species on the dung from ﻿each of﻿ the three different feeding guilds was visualized as bipartite network and analyzed using H_2_'^37^, representing a measurement for the overall specialization of the compositional dung type – beetle species network. The network analysis was performed with the R package “bipartite”^38^.

We statistically analyzed the C/N ratio, amount of amino/fatty acids [µg/mg], the water content [%] and the amounts of sterols [% Std./mg] of dung types (carnivore, omnivore, herbivore) using ANOVA and TukeyHSD post-hoc test. We checked for the normal distribution of the residuals and the homogeneity of variance prior to the analyses and transformed the data if necessary (C/N ratio, the amount of NLFAs s [µg/mg] were log-transformed, the amounts of sterols [% Std./mg] were log(x + 1)-transformed, the ratio of FFA/total lipids [%] was arcsine square root transformed whereas the amount of free fatty acids [µg/mg] and amino acids [µg/mg] were square root transformed and 4^th^-square root transformed, respectively). To compare the cholesterol/ sterol ratio [%] and the amount of FFAs [µg/mg] across dung types we used Kruskal-Wallis tests with subsequent pairwise U-tests corrected for false discovery rate^39^ in multiple comparisons.

Compositional data of amino- and NLFA profiles were analyzed using discriminant analysis of principal components (DAPC) and PERMANOVA/PERMDISP as implemented in the R packages “adegenet”^40^ and “vegan”^41^, respectively. DAPC is a powerful method to discriminate *a priori* assigned groups in a multivariate ordination of chemical compositional data^42^. It transforms the original data by PCA prior to the discriminant analysis (DA) and therefore values become uncorrelated. We retained 7 (for amino acids) and 5 (for NLFAs) PC-axes based on their Eigenvalues and the explained variance. We further used PERMANOVA^43^ with Bray-Curtis similarities^44^ to test if overall composition of either amino acids (all amino acids and essential amino acids) or NLFAs differed across dung types. In case of significant PERMANOVA, we used PERMDISP^45^ to distinguish between location/dispersion effects (see^46^ and Brückner and Heethoff^42^ for details) and to test whether the compositional stability of nutrients differed among dung types. All statistical analyses were performed with R version 3.3.1 “Bug in Your Hair”^47^. Finally, we correlated the total number of dung beetles (i.e. pooled from all plots) trapped on the respective dung type to different nutritional parameters of the dungs (means of C/N ratio, amounts of all/essential amino acids, NLFAs, FFAs and sterols as well as cholesterol/sterol ratio and water content) using Spearman’s rank correlation in PAST 3^48^.

## Results

### Nutritional analyses

**Figure 1:**
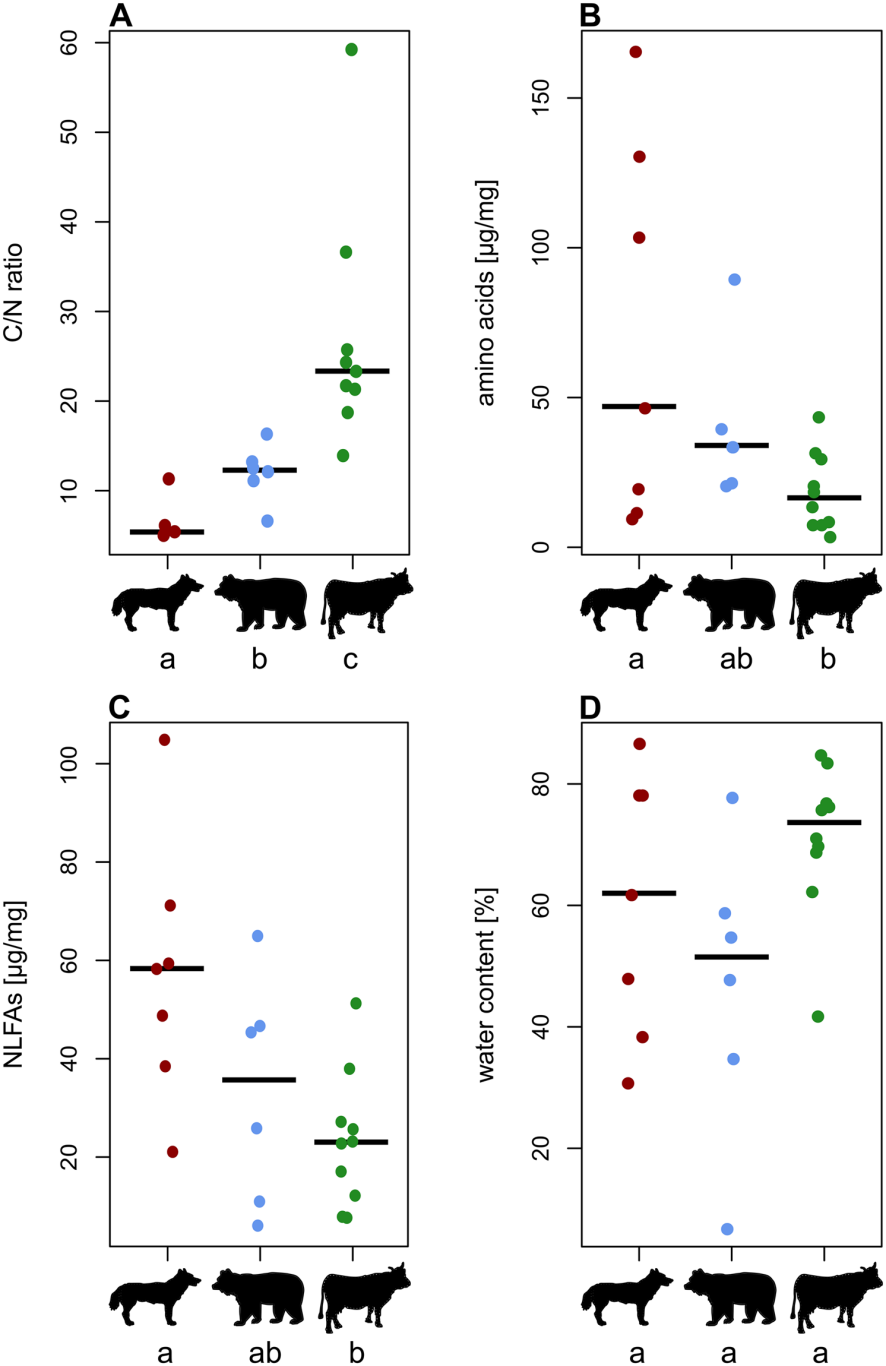
Nutritional values for carnivore, omnivore and herbivore dung (left to right): C/N ratio (**A**), amino acids (**B**), neutral lipid fatty acids - NLFAs (**C**) and water content (**D**). ﻿Each point represents a different vertebrate species﻿, and﻿ lines within each feeding guild represent the median. Significant differences among groups are marked by different letters for each panel. (Animal drawings in Figs 1–4 by Adrian Brückner).

**Figure 2:**
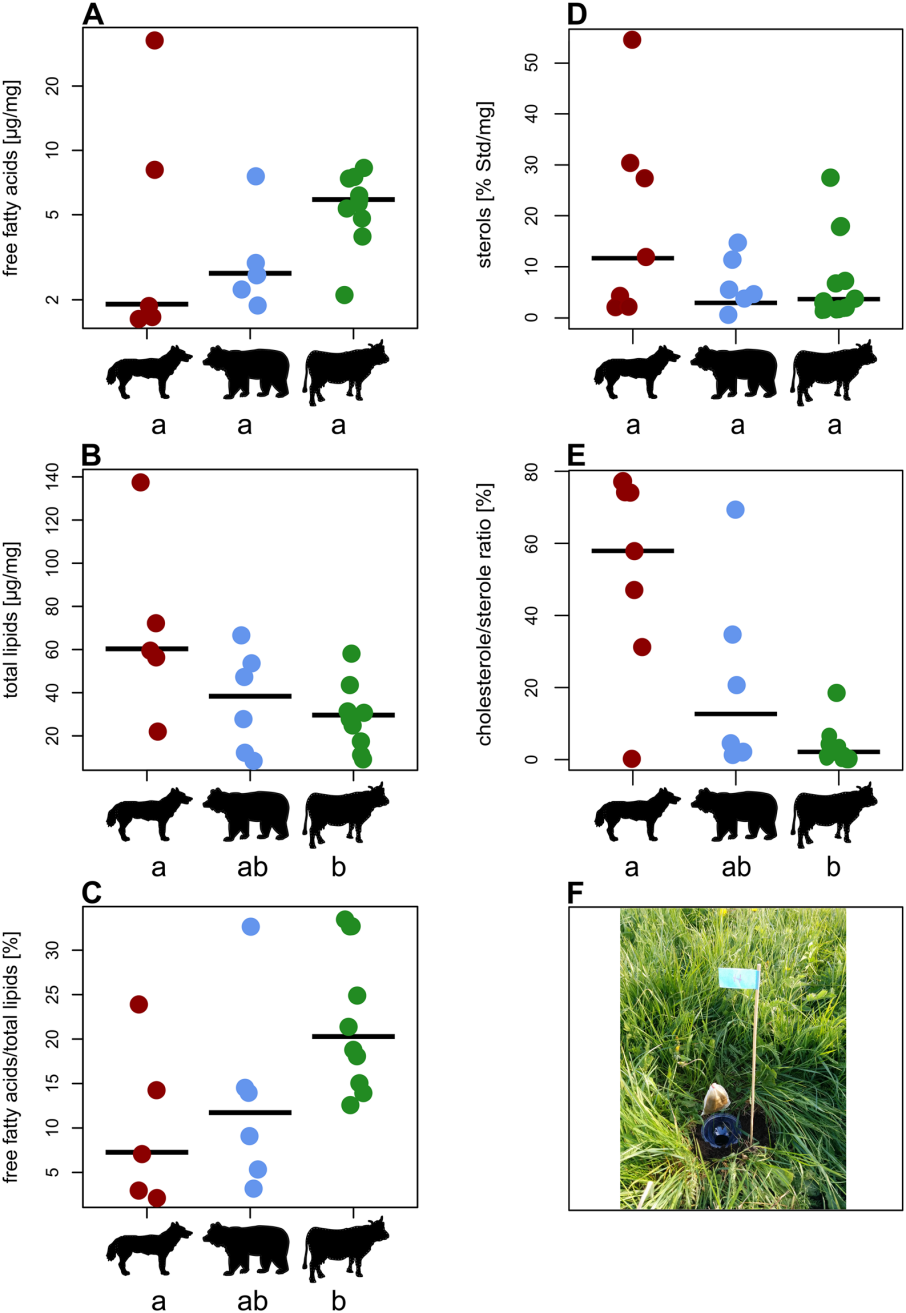
Nutritional values for carnivore, omnivore and herbivore dung (left to right): free fatty acids (**A**), total lipids (**B**), free fatty acids/total lipids ratio (**C**), sterols (**D**) and cholesterol/sterol ratio (**E**). Each point represents a different vertebrate species﻿, ﻿and lines within each feeding guild represent the median. Significant differences among groups are marked﻿ by different letters for each panel. (**F**) Shows a pitfall trap with dung bait for dung beetle sampling.

**Figure 3:**
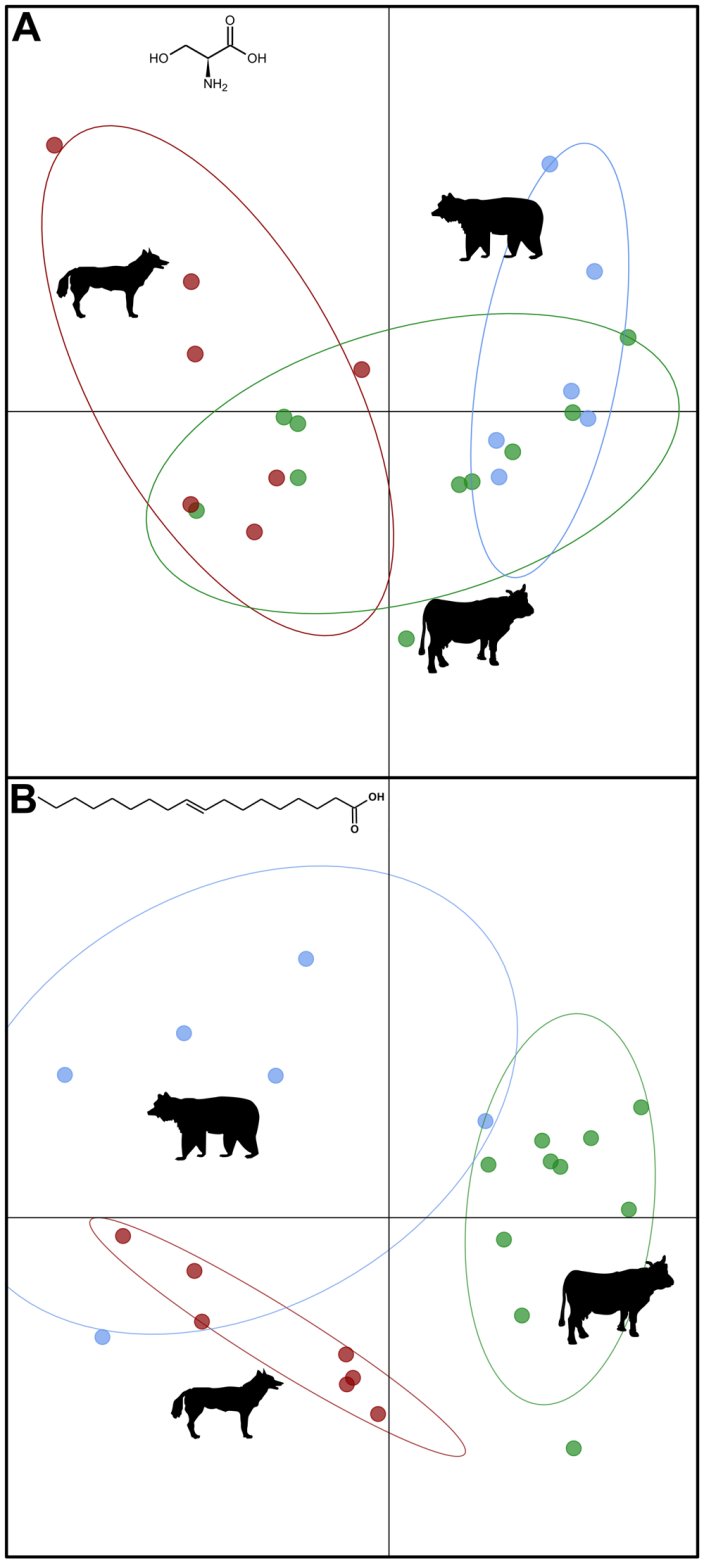
Discriminant analysis of principal components (DACP) for amino acids (**A**) and neutral lipid fatty acids - NLFAs (**B**). Feeding guilds are clustered in red for carnivore, in blue for omnivore and in green for herbivore dung.

Nutrients in vertebrate dung samples (see summarized in Table 1) differed significantly across the three feeding guilds (ANOVA; C/N ratio: F_2,18_ = 26.5, p < 0.001, Fig. 1A; amino acids: F_2,20_ = 3.7, p = 0.044, Fig. 1B; NLFAs: F_2,20_ = 4.3, p = 0.028, Fig. 1C), except for water content (F_2,20_ = 3.1, p = 0.065, Fig. 1D) and FFAs (Kruskal-Wallis test; χ^2^ = 2.6, d_f_ = 2, n = 21, p = 0.267, Fig. 2A). However, the total lipid content (NLFAs and FFAs combined) showed differences among the feeding guilds (ANOVA; F_2,18_ = 3.9, p = 0.039, Fig. 2B) and the amounts of FFAs and NLFAs were not correlated (Spearman´s rank: ρ_s_ = 0.14, p = 0.55). Also for the FFA/total lipid ratio and cholesterol/sterol ratio we found significant differences among the feeding guilds (FFAs/total lipid ratio: ANOVA; F_2,18_ = 4.4, p = 0.028, Fig. 2C; cholesterol/sterol ratio: Kruskal-Wallis test; χ^2^ = 8.2, d_f_ = 2, n = 23, p = 0.017, Fig. 2E), while sterol amounts were similar (ANOVA; F_2,20_ = 0.9, p = 0.44, Fig. 2D). The amino acids differed in relative composition between the feeding guilds (PERMANOVA; all amino acids: pseudoF_2,20_ = 2.7, R^2^ = 0.22, p = 0.013, Fig. 3A; only essential amino acids: pseudoF_2,20_ = 2.2, R^2^ = 0.18, p = 0.039) but not in overall dispersion across species within each guild (PERMDISP; all amino acids: F_2,20_ = 1.5, p = 0.25, Figs 3A, S2 II, Table 2; only essential amino acids: F_2,20_ = 1.1, p = 0.36), whereas NLFAs differed both in composition and dispersion (PERMANOVA; pseudoF_2,20_ = 4.8, R^2^ = 0.32, p < 0.001; PERMDISP: F_2,20_ = 5.0; p = 0.017, pairwise PERMDISP herbivore vs. omnivore p = 0.008; Figs 3B, S2 I, Table 3). In summary (see Table 1), carnivore dung types provided higher nutritional values (highest mean values for amino and fatty acids, sterols and cholesterol, lowest C/N ratio). Omnivore dung types provided intermediate nutritional value throughout the analyses.

**Table 1:** Nutrient parameters of vertebrate dung samples for the three feeding guilds; carnivores (n = 7), omnivores (n = 6) and herbivores (n = 10).

	C/N ratio	Amino acids [µg/mg]	NLFAs [µg/mg]	FFAs [µg/mg]	FFAs/total lipid ratio [%]	Water content [%]	Sterols [% Std./mg]	Cholesterol/ sterol ratio [%]
**Carnivores**								
lynx	5.4	166	105	33	24	62	27	47
mink	5.0	104	71	2	3	31	55	31
otter	5.4	47	49	8	14	78	1	77
raven	—	10	60	—	—	78	2	58
snowy owl	—	12	39	—	—	87	11	74
wildcat	6.1	131	21	2	9	48	30	0.3
wolf	11.3	20	58	2	3	39	1	74
mean	6.6 ± 2.4	70 ± 59	58 ± 24	9 ± 12	11 ± 8	61 ± 20	18 ± 19	52 ± 26
**Omnivores**								
brown bear	16.3	34	47	8	15	78	11	2
chicken	12.1	22	6	3	33	55	0.1	69
fox	6.6	40	26	3	10	48	2	21
gerbil	13.2	34	11	2	15	7	14	1
raccoon	11.1	90	65	2	3	59	3	35
wild boar	12.5	21	45	3	6	35	2	5
mean	11.9 ± 2.9	40 ± 23	33 ± 21	4 ± 2	14 ± 10	47 ± 21	5 ± 5	22 ± 24
**Herbivores**								
cow	21.3	21	17	8	32	84	7	19
deer	13.9	30	38	6	14	63	2	4
donkey	59.2	4	27	5	16	76	3	1
elephant	23.3	8	51	8	14	77	3	2
elk	24.3	14	12	6	33	71	26	0.2
goat	25.7	8	8	2	20	42	6	2
horse	36.6	9	23	5	18	76	2	4
rabbit	23.4	44	8	4	33	69	2	1
sheep	18.7	32	26	6	19	70	17	1
wisent	21.7	19	23	8	26	85	4	6
mean	26.8 ± 12.1	19 ± 12	23 ± 13	6 ± 2	21 ± 8	71 ± 12	7 ± 7	4 ± 5

The mean (±standard deviation) of the different nutrient parameters are shown for each feeding guild. NLFAs = neutral lipid fatty acids, FFAs = free fatty acids; – no measurement.

**Table 2:** Free and protein-bounded amino acids [in %] for each dung type, sorted by feeding guild (type: C = carnivore, O = omnivore, H = herbivore).

species	type	amino acid [%]																
		OH-Pro	Thr	Ser	Glu	Pro	Gly	Ala	Val	Cys	Met	Ile	Leu	Tyr	Phe	Lys	His	Arg
lynx	C	1.8	5.4	9.5	11.7	8.5	18.3	14.1	5.3	1.2	1.0	2.3	7.1	1.8	2.6	5.3	1.5	2.7
mink	C	—	5.9	10.9	9.6	7.6	16.4	13.1	3.8	—	—	2.7	14.0	3.1	2.9	6.2	0.9	3.0
otter	C	—	9.6	13.3	10.1	9.9	16.5	14.7	3.7	—	—	1.2	9.7	2.1	2.1	4.2	1.0	1.8
raven	C	—	5.9	9.8	13.1	8.4	18.4	13.1	3.7	0.5	1.3	2.6	8.3	1.9	2.0	6.1	2.2	2.7
snowy owl	C	—	5.1	13.6	12.1	9.6	15.5	9.8	4.2	2.6	1.2	3.0	7.9	2.3	3.5	4.4	1.5	3.6
wildcat	C	3.7	4.4	8.4	12.3	10.2	23.1	11.3	3.3	0.4	1.2	1.9	6.0	1.4	2.2	5.4	2.1	2.7
wolf	C	1.1	6.0	10.3	12.3	8.6	17.2	11.4	5.6	0.9	1.3	2.7	7.5	1.8	2.9	5.4	2.0	3.0
brown bear	O	0.5	5.6	8.3	12.5	6.6	16.1	16.3	5.8	—	1.7	2.8	8.0	2.0	3.1	7.3	1.5	1.9
chicken	O	—	5.1	7.0	9.0	9.8	19.9	17.5	2.3	—	0.5	2.5	10.7	1.5	2.8	8.6	0.7	2.2
fox	O	0.7	4.6	8.0	10.6	7.0	20.7	16.4	3.9	—	1.2	2.8	8.2	1.5	2.9	8.0	1.7	1.7
gerbil	O	—	5.4	8.0	11.7	7.1	17.4	17.8	4.2	—	1.4	2.8	8.6	1.9	2.9	7.5	1.5	2.0
raccoon	O	—	5.5	7.4	11.6	7.1	19.9	18.9	2.0	—	—	2.2	10.7	1.6	2.5	8.2	0.7	1.6
wild boar	O	1.2	5.0	8.2	13.2	6.3	17.6	17.7	3.7	—	1.5	2.6	9.9	1.6	3.0	5.3	1.4	1.8
cow	H	0.2	5.2	7.8	11.1	8.1	17.4	16.1	3.6	—	1.2	2.7	10.1	1.9	3.2	8.0	1.3	2.1
deer	H	—	5.7	8.3	12.9	6.9	15.8	16.7	4.1	—	1.6	2.7	9.1	2.0	3.3	7.2	1.4	2.1
donkey	H	—	4.8	10.8	12.8	7.5	16.2	10.9	4.3	3.5	1.3	2.9	8.2	2.9	3.0	6.1	1.4	3.3
elephant	H	0.1	4.4	14.8	9.9	10.3	16.8	9.3	4.7	2.9	0.9	3.1	7.6	3.1	4.0	3.1	1.1	3.8
elk	H	—	4.6	7.3	11.6	7.5	19.6	17.3	2.7	—	0.8	2.3	9.8	1.6	2.9	8.3	1.5	1.9
goat	H	2.6	3.5	6.2	12.2	8.1	19.8	14.8	4.9	0.2	1.0	2.9	7.6	1.8	3.0	7.6	1.3	2.5
horse	H	1.6	5.3	8.0	12.5	8.8	18.3	13.9	6.1	0.2	1.1	2.6	7.2	1.6	2.7	6.3	1.6	2.1
rabbit	H	—	6.4	7.6	9.9	7.5	17.5	20.1	3.1	—	1.0	2.3	9.4	1.7	2.5	8.0	1.4	1.8
sheep	H	0.1	6.1	10.3	13.3	8.6	17.2	11.7	4.2	0.9	1.5	2.7	8.4	2.0	2.4	5.3	2.3	3.0
wisent	H	1.8	4.8	8.3	13.0	9.3	20.5	13.2	3.2	0.1	1.3	2.2	7.4	1.4	2.7	6.2	2.1	2.5

**Table 3:** Neutral lipid fatty acids [in %] derived from dietary saturated, mono- and polyunsaturated fats for each dung type, sorted by feeding guild (type: C = carnivore, O = omnivore, H = herbivore; ^§^ = double bond posit﻿ion not further﻿ determined).﻿

species	type	fatty acids derived from dietary fats [%]																				
		saturated												monounsaturated					polyunsaturated			
		C10	C12	C14	C15	C16	C17	C18	C20	C22	C24	C26	C28	C16:1Δ7	C16:1Δ9	C18:1Δ9	C18:1Δ10	C18:1Δ11	C18:2Δ9,12	C18:2^﻿§﻿^	C20:4Δ5,8,11,14	C20:3Δ8,11,14
snowy owl	C	0.6	0.9	3.0	1.2	43.2	0.3	41.1	—	—	—	—	—	—	1.0	5.5	0.8	—	2.4	—	—	—
lynx	C	0.2	0.1	1.8	0.7	39.8	1.2	44.7	0.3	0.2	0.2	—	—	—	0.3	8.4	0.6	0.7	0.6	0.1	0.1	—
mink	C	0.1	—	0.2	—	39.3	0.3	17.9	0.3	0.1	0.1	—	—	0.1	0.2	40.7	—	—	0.7	0.1	—	—
otter	C	0.2	0.2	1.4	0.3	55.5	0.3	18.6	—	—	—	—	—	—	1.0	11.5	1.1	—	7.8	2.2	—	—
raven	C	0.3	0.6	1.7	0.4	35.5	0.8	42.6	0.5	—	—	—	—	—	0.8	9.7	1.5	—	4.1	1.0	0.6	—
wildcat	C	—	0.1	0.7	2.6	71.2	0.4	20.3	0.2	0.3	0.3	—	—	0.2	0.2	0.7	0.8	1.2	0.7	0.1	—	0.1
wolf	C	0.1	0.1	2.0	0.8	47.3	1.7	40.1	0.3	0.1	0.1	—	—	0.1	0.4	4.3	0.8	1.1	0.7	0.1	—	0.1
rabbit	H	0.1	0.3	1.0	1.3	35.5	0.4	28.4	0.4	0.5	0.3	0.3	0.3	—	0.8	1.9	0.5	—	5.7	22.5	—	—
elk	H	0.4	1.0	3.9	4.3	33.7	3.8	38.8	0.9	0.9	1.1	0.8	1.1	—	—	2.4	3.6	—	2.1	0.6	—	—
elephant	H	0.1	0.4	2.0	2.1	20.3	0.7	58.4	0.8	0.6	0.3	0.2	0.2	—	—	1.8	1.6	8.4	0.9	0.9	—	—
donkey	H	0.1	0.6	7.0	7.4	33.4	2.2	38.1	0.5	0.7	0.6	0.9	0.5	—	0.1	1.1	3.3	—	0.9	0.3	—	-
cow	H	0.5	0.9	3.7	2.3	32.7	2.3	46.8	0.4	0.5	0.6	0.4	0.5	—	0.1	1.9	1.9	2.6	0.6	—	—	—
horse	H	0.2	1.0	5.7	6.2	36.2	1.7	37.2	0.8	0.6	0.6	0.5	0.4	—	—	1.7	5.2	—	0.8	—	—	—
deer	H	0.2	0.4	1.7	1.9	22.6	1.5	63.6	0.6	0.4	0.4	0.5	0.0	—	—	1.6	3.1	—	0.9	0.2	—	—
sheep	H	0.2	0.9	2.9	4.0	27.5	2.4	48.0	0.7	0.5	1.2	1.6	0.4	—	—	1.6	2.9	3.2	0.7	0.2	—	—
wisent	H	0.3	0.8	2.5	2.0	26.4	1.8	55.7	0.6	0.5	0.5	1.5	0.5	—	—	1.6	0.7	2.5	0.9	0.2	—	—
goat	H	0.5	1.6	4.8	3.8	33.7	2.9	37.1	1.3	1.1	1.0	2.9	2.3	—	—	2.1	0.7	—	2.4	0.7	—	—
brown bear	O	0.4	1.6	4.7	3.3	33.3	2.8	37.0	1.2	0.9	0.8	2.2	1.4	—	—	2.5	1.3	0.7	4.1	0.5	—	—
chicken	O	1.2	1.2	1.5	0.1	81.8	0.2	9.7	0.1	0.1	0.1	—	—	—	—	2.4	0.3	—	1.1	0.1	—	—
fox	O	0.1	0.3	2.0	0.7	41.9	0.4	15.8	0.3	0.3	0.3	—	—	—	4.8	24.7	3.9	1.6	2.2	0.8	—	0.1
gerbil	O	—	0.3	1.0	0.9	32.1	0.2	14.6	0.8	0.3	0.2	0.2	—	—	0.1	36.3	0.4	—	11.6	0.9	—	—
racoon	O	—	—	0.1	—	12.3	—	4.8	0.7	1.6	0.4	—	—	—	—	77.4	—	—	1.8	0.4	0.2	0.2
wild boar	O	0.1	0.2	1.4	2.3	27.7	1.1	39.4	0.6	0.3	0.2	0.1	—	—	0.2	8.5	1.5	10.7	4.3	1.5	—	—

### Dung type preference

**Figure 4:**
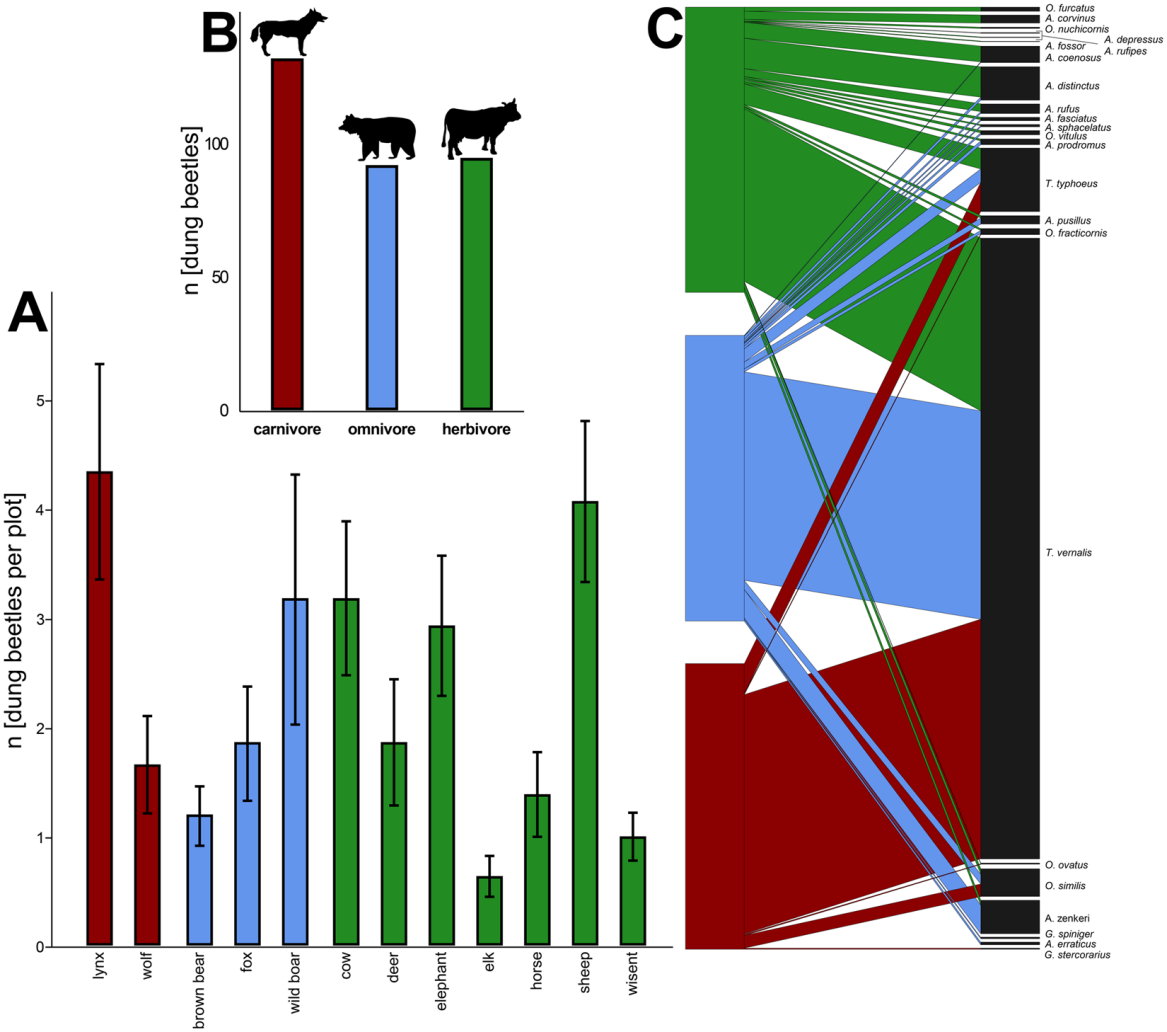
Overview of trapped beetles for a subset of 12 representative dung types offered in 27 forests and 27 grasslands. (**A**) shows the mean number of trapped beetles for each dung type (red = carnivore, blue = omnivore, green = herbivore), while (**B**) shows the total number of trapped beetles per feeding guild. (**C**) A resource - beetle trophic network shows the proportional distribution of dung beetle species across dung from three feeding guilds (red = carnivore, blue = omnivore, green = herbivore dung).

We sampled a total of 1191 individuals from 23 dung beetle species in 40 sites; in 14 out of 54 sites no dung beetles were trapped. Overall, dung beetles were attracted to all 12 dung types offered (Fig. 4C). Species-specific preferences of the beetles among dung types were significant (Friedman test: χ^2^ = 62.1, d_f_ = 11; n = 648, p < 0.0001, Fig. 4A), but not consistent across feeding guilds (Friedman test: χ^2^ = 2.8, d_f_ = 2, n = 162, p = 0.25, Fig. 4B). Different beetle species had relatively similar preferences and showed no clear species partitioning across dung types, hence there was only a relatively low degree of complementary specialization in the dung type – beetle network (H_2_′ = 0.30, 23 beetle species across dung from 3 feeding guilds).

### Correlation between dung nutrients and attractiveness for dung beetles

Overall, there was no correlation between dung beetle abundances and any of the nutrient parameters for all 12 dung types (Spearman’s rank: C/N ratio: ρ_s_ = −0.48, p = 0.11; all amino acids: ρ_s_ = 0.46, p = 0.14; essential amino acids: ρ_s_ = 0.42, p = 0.17; NLFAs: ρ_s_ = 0.40, p = 0.20; FFAs: ρ_s_ = 0.16, p = 0.62; water content: ρ_s_ = −0.36, p = 0.24; sterols: ρ_s_ = 0.15, p = 0.55, cholesterol/sterol ratio: ρ_s_ = 0.26, p = 0.41).

## Discussion

Dung beetles strongly depend on a resource that is scarce and patchy in occurrence. Yet, dung is immobile as well as chemically and mechanically undefended, which makes it an easily acquirable and valuable source of energy. Adult dung beetles are attracted to many different dung types, regardless of the type of dung on which they have fed and grown as a larva^7,14,33^. Furthermore, especially in the tropics, some of these beetles became highly specialized in resource usage^49^.

Our field experiment demonstrated a generalized usage of all offered dung types, but also a significant difference in the quantity of beetles attracted across dung types (Fig. 4A–C). Generally, higher amounts of dung attract more dung beetles^50^, but since we used equal amounts of dung for the baits (approx. 35 g each) there must be alternative explanations for their preferences, for which the amount and composition of volatile organic compounds must play a key role^16,51^ given that the beetles had no contact to the dung in our experiment (see Fig. 2F). For instance, indole and skatole, two weak/moderate attractors^51^ are molecules derived from the decomposition of the amino acid tryptophan, whereas phenolic compounds (e.g., phenol, p-cresol, p-ethyl phenol) are derivatives of phenylalanine and tyrosine. Also, fatty acids and fatty acid derived compounds like butyric acid, unspecific butanones and butanoles or ethyl-/butyl-esters are present in various dungs^33,52^. Hence, dung odour bouquets as proximate cues may potentially include intrinsic information on their ultimate cause, i.e. dung nutrients (especially amino- and fatty acids) which are converted and rearranged to volatile organic compounds.

Accordingly, besides the attractive function of dung volatiles^33,51,52^, these compounds may also serve as “nutritional cues” for dung beetles. Therefore we asked whether the beetles’ preference matched the differences in nutrient quality of dung. Although beetles used all dung types, some were preferred over others, and the most preferred ones occurred across the feeding guilds (e.g. lynx, wild boar and sheep). Nutritional composition, however, was no significant predictor to explain the beetle’s preference. Hence, volatiles are most probably no nutritional cues. Yet, volatile organic compounds only recently receive growing attention in dung beetle research^16,17,33,51,53^ and more in-depth analyses may help to unravel the beetles’ resource choices linked to dung nutrients^52^.

In general, animal droppings vary in nutrient amounts, even within a species or feeding guild^54^. Whereas higher nutrient concentrations are generally beneficial, dung beetles may face trade-offs that constrain a higher preference of nutrient-rich dung. Carnivore dung, for example, is more nutrient rich compared to herbivore dung (Figs 1–3 ^14^), but could contain pathogenic bacteria, which are perceived by the dung beetles via olfactory cues^53^.

The C/N ratio is frequently used as an index for quality descriptions of organic substrates including dung^54^. In general C/N ratios increased over ten-fold from carnivore dung (lowest value for mink, 5.0) to herbivore dung (highest value for donkey, 59.2); omnivore dung has intermediate levels (Fig. 1, Table 1). Corresponding to a higher nitrogen (N) content (i.e. the reverse of the C/N ratio), the amount of amino acids and thus a higher nutritional value^55^ increased from carnivore to herbivore dung, associated with a change in proportional composition of amino acid (Fig. 3). Still, all dung types contained nearly all amino acids that are essential for insects and thus for dung beetles^56^ (except for methionine in mink, otter and raccoon dung). Amino acids play key roles in insect development such as the emergence from the pupal skin, they are precursors of pigments or promote growth in larvae and adults^57^. Therefore our results highlight that dung is able to supply the beetles’ need for amino acids in general – for further synthesis as well as for direct use.

Similar to the trend in nitrogen and amino acids, the amount of NLFAs increased over ten-fold from herbivores (goat and rabbit, 8 µg/mg) via omnivores to carnivores (lynx, 105 µg/mg). Additionally, feeding guilds had specific fatty acids corresponding to their food such as unsaturated fatty acids (e.g. oleic and linoleic acid) for carnivores and omnivores, while herbivores showed higher amounts of saturated fatty acids (Table 3). Interestingly, there was no analytical indication for α-linolenic acid (C18:3Δ9, 12, 15) in NLFAs and only in trace amounts for FFAs. α-linolenic acid is thought to be one of the essential fatty acids for insects^58^ and hence, needs to be consumed or supplemented by symbiotic bacteria. Although our method allows for detecting FFAs from C6:0 (caprylic acid) to C28:0 (montanic acid), we only found C16:0/C18:0 in notable amounts. These amounts were similar among all feeding guilds and also the overall lipids [µg/mg] showed a similar pattern to NLFA amounts. However, the FFAs/total lipid ratio (Fig. 2C) indicated more free fatty acids in herbivore than in carnivore dung, suggesting different performance in fat digestion in these vertebrates but also different palatability for dung beetles and other coprophagous animals. Overall, the high concentrations of fatty acids and other nutrients supports the view that dung is not waste, but a valuable resource for coprophagous beetles^6^, which particularly utilize fatty acids during growth and larval emergence^59–61^.

Water contents of different dung types were similar (except for gerbil dung that contained only 7% water) (Fig. 1, Table 1). Still, water content plays an important role, as adult dung beetles mainly use the liquid phase and its nutrients/particles to feed on^27^, it affects the occurrence of species^15^ and the handling for brood balls^62^.

Insects, unlike other animals, lack the ability to synthesize sterols, and they must obtain such compounds via food or bacterial symbionts^56^. Sterols have several key functions, since they serve as components of the cell-membrane (especially cholesterol), as regulators of developmental genes and as precursors of different hormones^63^. Our analysis confirmed sterols and cholesterol in all dung types, yet some amounts were extremely low, especially in herbivores. Carnivore dung seems to be a valuable resource regarding cholesterol (Fig. 2D–E), which typically ﻿needs to be metabolized from certain phytosterols by herbivores﻿^64–67^. Dung beetles may thus either obtain cholesterol directly from dung (especially carnivore dung) or synthesize it from sterols in herbivore/omnivore dung. The amount of sterols in dung may, however, be too low to fully supplement dung beetles, thus consumption of other food (e.g. plant material^12^) or bacterial symbiosis might help to acquire all mandatory sterols.

## Conclusion

All nutrient parameters, C/N ratio, amino acids, fatty acids, cholesterol/sterol ratio and the composition of amino acids varied across dung types and feeding guilds. Although dung represents an already-digested, but still valuable resource it grants sufficient amounts of most (essential) nutrients for insects. Hence, symbiotic bacteria may not be mandatorily needed for nutritional upgrading. Regarding C/N, protein (=amino acid content) and fatty acids (=NLFAs and free fatty acids), dung showed similar values to resources available for other terrestrial beetles, such as litter, fruits, fungi and carcasses (see Supplementary Information; Table S3). We did not confirm that nutritional composition drives the beetles’ food selection, suggesting that the beetles’ attraction to specific blends of volatiles are uncoupled from nutrient values, and thus volatiles may not serve as nutritional cues.

### Ethics statement

Field work and animal collection permits were issued by the responsible state environmental offices of Baden-Württemberg, Thüringen, and Brandenburg (according to § 72 BbgNatSchG).

### Data, code and materials

All datasets supporting this article have been uploaded as part of the Supplementary Material.

## Electronic supplementary material

Supplementary Information
