# Supplementary material for: Nutrient quality of vertebrate dung as a diet for dung beetles
Source: Sci Rep. 2017 Sep 22;7:12141. doi: 10.1038/s41598-017-12265-y (PMC5610319; doi:10.1038/s41598-017-12265-y)
Supplement: Supplementary file 1 — Supplementary Information [file 41598_2017_12265_MOESM1_ESM.pdf]

Supplementary Information:
Nutrient quality of vertebrate dung as a diet for dung beetles
by Kevin Frank, Adrian Brückner, Andrea Hilpert, Michael Heethoff and Nico Blüthgen
Corresponding author: bluethgen@bio.tu-darmstadt.de

Table S1: Food plan of the animals whose dung was used in this study

species	origin	diet	feeding guild
lynx	wildlife park 'Alte Fasanerie'	meat of cattle, chicken, rabbits, pigeon and deer	carnivore
mink	zoo 'Opel-Zoo'	meat of chicken, mice, rats, pigeon and freshwater fish	carnivore
otter	zoo Vivarium	freshwater fish, rats, mice	carnivore
raven	zoo Vivarium	rats, mice	carnivore
snowy owl	zoo Vivarium	rats, mice	carnivore
wildcat	zoo 'Opel-Zoo'	meat of chicken, mice, rats, pigeon and freshwater fish	carnivore
wolf	wildlife park 'Alte Fasanerie'	meat of cattle, chicken, rabbits, pigeon and deer	carnivore
brown bear	Fasanerie Wiesbaden	vegetables, fruits, occasionally meat and fish	omnivore
chicken	private stock	wheat, maize, seeds, salad, grass, vegetables, mealworms, eggshells and chalk (grit)	omnivore
fox	wildlife park 'Alte Fasanerie'	60 % meat (chicken, mice, rats, cattle), fruits, vegetables	omnivore
gerbil	private stock	mealworms, carrots, seed mixture (oat, linseed, lucerne)	omnivore
raccoon	zoo 'Opel-Zoo'	fruits and vegetables, eggs; occasionally meat	omnivore
wild boar	wildlife park 'Alte Fasanerie'	pig food (raiffeisen), bread, maize, fruit, vegetables, lucerne pellets, meat of cattle & deer and red deer	omnivore
cow	Oberfeld farm (organic)	grazing on pasture, hay	herbivore
deer	wildlife park 'Alte Fasanerie'	grass, hay, maize, fodder beet, lucerne pellets, apples, carrots	herbivore
donkey	zoo Vivarium	fresh and dried fruits, hay silage, fresh hay, grazing on pasture, maize, barley, oat	herbivore
elephant	zoo 'Opel-Zoo'	grass, hay, oat, wheat bran, apples, carrots, branches; occasionally lucerne hay, mineral briquet	herbivore
elk	zoo 'Opel-Zoo'	lucerne hay, moose-pellets (mazuri), leaves and bark	herbivore
goat	zoo Vivarium	hay silage, fresh hay, grazing on pasture, maize	herbivore
horse	Oberfeld farm (organic)	grazing on pasture	herbivore
rabbit	private stock	carrot, salad, grass, herbs	herbivore
sheep	Oberfeld farm (organic)	grazing on pasture	herbivore
wisent	wildlife park 'Alte Fasanerie'	grazing on pasture, hay	herbivore

Supplementary Methods S1: Detailed methods of dung sampling and chemical analyses

Dung used in this study

We used 23 different dung types of carnivorous, omnivorous and herbivorous species, namely: brown bear (Ursus arctos L., 1758), chicken (Gallus gallus domesticus L., 1758), cow (Bos taurus L., 1758), donkey (Equus africanus asinus L., 1758), elephant (Loxodonta africana Blumenbach, 1797), elk (Cervus canadensis Erxleben, 1777), fox (Vulpes vulpes L., 1758), gerbil (Meriones unguiculatus Milne-Edwards, 1867), goat (Capra aegagrus hircus L., 1758), horse (Equus caballus L., 1758), lynx (Lynx lynx L., 1758), mink (Mustela lutreola L., 1761), otter (Aonyx cinerea Illiger, 1815), rabbit (Oryctolagus cuniculus domestica L., 1758), raccoon (Procyon lotor L., 1758), raven (Corvus corax L., 1758), red deer (Cervus elaphus L., 1758), sheep (Ovis aries L., 1758), snowy owl (Bubo scandiacus L., 1758), wild boar (Sus scrofa L., 1758), wild cat (Felis silvestris silvestris Schreber, 1777), wisent (Bison bonasus L., 1758) and wolf (Canis lupus L., 1758). Game species´ and domestic animals´ dung was collected in wildlife parks, zoos or came from private stocks. Since the captive animals' diet may differ from those of wild animals, we collected our dung samples only from organic farms and wildlife parks / zoos where the animals are kept on most natural diets without food additives and concentrated feeding stuff. The detailed food plan can be found in supplementary table S2.

Dung sampling in the Biodiversity Exploratories

For dung beetle field samplings we used pitfall traps equipped with the dung baits of 12 different representative subsamples (i.e. dung available in sufficient amounts) collected form mammal species, namely: wolf, lynx, fox, brown bear, wild boar, cow, horse, sheep, deer, elephant, elk and wisent (2 carnivores, 3 omnivores and 7 herbivores). The traps were set up randomized on a transect, in a total of 54 experimental sites (27 in forests, 27 in grasslands) in three regions of Germany. The regions are: (1) Biosphere Reserve Schorfheide-Chorin (in North-East Germany, ~13.000 km2, 3 – 140 m a.s.l., 13°23'27''–14°08'53'' E / 111

52°47’25’’–53°13’26’’ N), (2) Hainich National Park and surroundings (in Central Germany, ~13.000 km2, 285 – 550 m 112 a.s.l., 10°10’24’’–10°46’45’’ E / 50°56’1 4’’–51°22’43’’ N) and (3) Biosphere Reserve Schwäbische Alb (in South-West Germany, ~422 km2, 460 – 860 m a.s.l., 09°10’49’’–09°35’54’’ E / 114 48°20’28’’– 48°32’02’’ N).

Lipid analyses

Total neutral lipids (hereafter, neutral lipid fatty acids = NLFAs) were extracted from the fresh dung samples (40 - 50 mg of fresh weight) using 1 ml of a chloroform:methanol-mixture, 2:1 (V/V) over a period of 24 h. Afterwards extracts were purified and separated using SiOH-columns (Chromabond® SiOH, Macherey-Nagel GmbH & Co. KG, Düren, Germany) which were washed and conditioned with 6 ml hexane. Afterwards samples were applied on the column and elution of neutral lipids was accomplished with 4 ml of chloroform. Afterwards the chloroform fraction was evaporated to dryness under nitrogen gas flow and residuals were redissolved in dichloromethane:methanol, 2:1 (V/V) using different amounts of solvent depending on the dung-type (200 µl for herbivores, 500 µl for omnivores and 750 µl for carnivores) to adjust the concentration. 20 µl were transferred to chromatographic glass vials with a conical inlet (150 µl), 20 µl nonadecanoic acid (220 ng/µl) was additionally added as internal standard and the mixture was evaporated to dryness again and subsequently derivatized to fatty acid methyl esters (FAMEs) with TMSH (trimethylsulfonium hydroxide; 0.25 M in MeOH from Fluka, Sigma-Aldrich, St. Louis, USA) reagent according to the supplier’s information. Free fatty acids (= FFAs) were extracted as described for NLFAs (see above), however we had to exclude raven and snowy owl from this analysis. Afterwards extracts were purified and separated according using SiOH-columns (Chromabond® SiOH, Macherey-Nagel GmbH & Co. KG, Düren, Germany) which were washed and conditioned with 6 ml hexane. Afterwards samples were applied on the column and triglyceride/sterol- and diglyceride-fractions where eluted with 4 ml isooctane:ethyl acetate (10:1, V/V) and 4 ml isooctane:ethyl acetate (3:1, V/V), respectively

and discarded afterwards. Free fatty acids were finally eluted with 4 ml of a solvent mixture containing isooctance:ethyl acetate:acetic acid (75:25:2; V/V/V). Subsequently the final solvent fraction was evaporated to dryness under nitrogen gas flow and residuals were redissolved in dichloromethane:methanol, 2:1 (V/V) and further processed as described above for NLFAs.

Amino acids

For analysis of the amino acids (free amino acids and protein-bounded), 5 mg (± 0.1 mg) dried dung was diluted in 200 µL of hydrochloric acid (6 mol/l) and boiled for four hours at 100°C, and cooled to room temperature afterwards. Note that this acidic chemical extraction decays asparagine, glutamine and tryptophan. Afterwards residuals were cooled to room temperature, centrifuged (10 min at 14,800 rpm) and the supernatants were transferred into fresh tubes and were evaporated to dryness at 100°C, before the samples were re-dissolved in 200 µL of deionized water and evaporated repeatedly. Samples subsequently were re-dissolved again in 200 µL of deionized water and finally, the amino acids were measured with an ion exchange chromatograph with ninhydrin post-column derivatization (Biochrom 20+, Amino Acid Analyzer, Cambridge, UK). A standard amino acid mixture (Laborservice Onken GmbH, Gründau, Germany) was used as external standard. The amount of total amino acids [µg] was standardized using the dry weight [mg] of the initial sample.

Table S2: Variation of nutrient concentrations within each dung type (sampled at different times and/or locations) expressed as coefficient of variation (CV = sd/mean). Mean values across all dung types shown for within-dung type variability (CV_{within}) and across-dung type variability (CV_{across}). In parentheses, range of CVs (min – max) shown across different dung types (^{D}) or different substances (^{S}). Nutrient variation across dung types was 3.4 to 7.6-fold higher across dung types than within. The mean number of replicates per dung type (*n*) is provided and its range in parentheses; CVs were calculated for all dung types where *n* > 1.

	n	CV_{within}	CV_{across}
Total amino acids	4 (2 – 10)	0.26 (0.03 - 0.67)^{D}	1.11
Single amino acids (14 substances)	4 (2 – 10)	0.30 (0.23 - 0.41)^{S}	1.15 (0.76 - 1.44)^{D}
Fatty acids and sterols (24 substances)	1.8 (1 – 4)	0.49 (0.13 - 0.92)^{S}	1.66 (0.60 - 3.19)^{D}
C/N ratio	5.1 (3 – 15)	0.09 (0 - 0.37)^{D}	0.71
Water content	2 (1 – 4)	0.10 (0 - 0.72)^{D}	0.34

Figure S1: Discriminant analysis of principal components (DACP) for and neutral lipid fatty acids, including all batch replicates. Groups are clustered in red for carnivore, in blue for omnivore and in green for herbivore dung. An additional cluster in pink shows cow dung from 11 different farms.

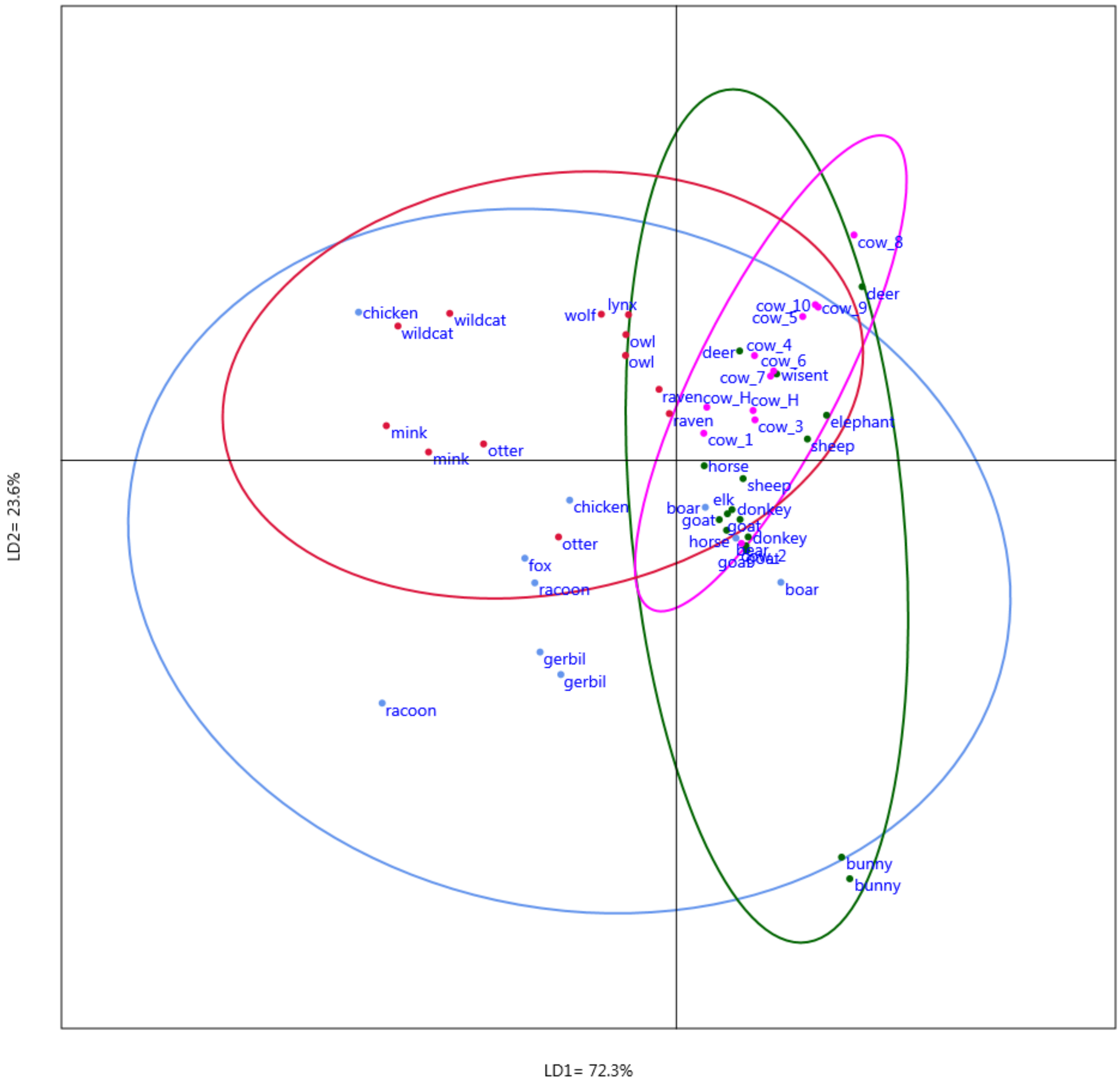

List S1: Linear regression formulas for dung dry weight calculation

Note: Always three point calibration, R^2=0.98-0.99

1 Cow: dw = 0.1764 x fw – 0.6322

2 Fox: dw = 0.4598 x fw + 2.4886

3 Wild boar: 0.6617fw-0.3902

4 Deer: dw= 0.4057fw-1.2012

5 Horse: dw=0.2549fw-0.7973

6 Sheep: dw=0.2881fw+0.5172

7 Lynx dw=0.3845fw+0.0083

8 Elephant dw=0.2608fw-1.3293

9 Elk dw=0.2897fw-0.1194

10 Wisent dw=0.2132fw-2.5741

11 Wolf dw=0.6352fw-0.8341

12 Bear dw=0.2397fw—0.98

13 Raven dw=(0.733fw+6.8379)*(100/27) # with correction for non-organic material

14 Otter dw=0.5547fw-16.707

15 Owl dw=0.6098fw+13.627*(100/16) # with correction for non-organic material

16 Donkey dw=0.1194fw+6.1563

17 Goat dw=0.5295fw+1.3208

18 Gerbil dw=0.879fw+4.1493

19 Bunny dw=0.2872x+1.9051

20 Chicken dw=0.5277fw-5.7143

21 Mink dw=0.4218fw+24.672

22 Racoon dw=0.461fw-4.5469

23 Wildcat dw=0.4484fw+6.4164

Figure S2: Dispersion for fatty acids (I) and amino acids (II) in carnivore, omnivore and herbivore dung samples.

I - Dispersion – fatty acids

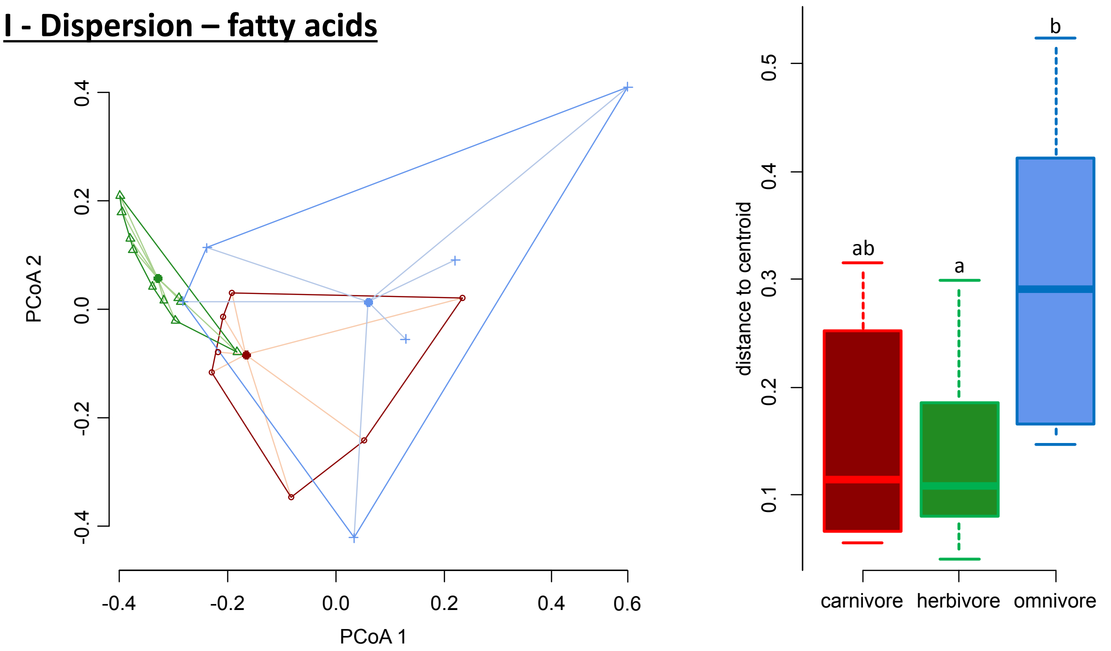

II - Dispersion – amino acids

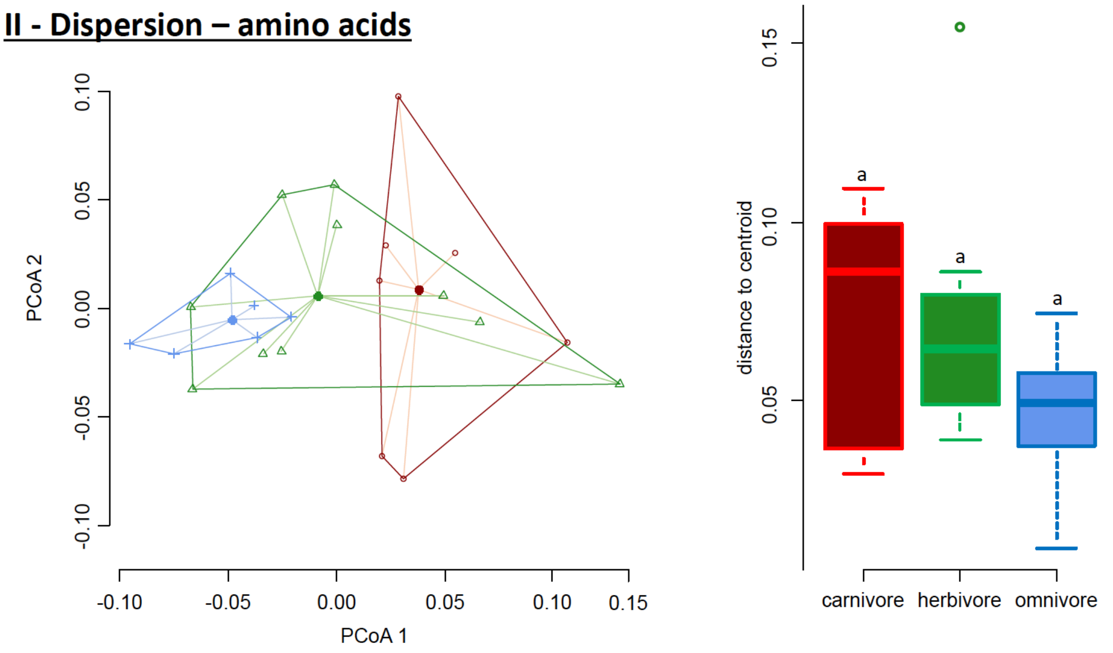

Table S3: C/N ratios, protein and fat content of other food sources potentially used by dung beetles.

Substrate	**C/N ratio**	**Protein [µg/mg]**	**Fat [µg/mg]**	**Reference**
leaf litter	40	13	5	Bridson (1985), Aerts (1997), Rawlins et al. (2006)
conifer litter	62	-	8	Bridson (1985), Aerts (1997)
apple fruit	31	3	4	Khudzari et al. (2016), Council directive: 90/496/EEC
fungi	8	109	15	Longvah and Deosthale (1998), Sadler (2003), Mouginot et al. (2014)
pig cadaver	8	180	150	Enser et al. (1996), Carter et al. (2007)
bone meal	4	480	80	Gotaas (1956), Hendriks et al. (2002)
dung beetle body	4	544	136	Raksakantong et al. (2010), Blüthgen unpublished

References

Aerts, R., 1997. Climate, Leaf Litter Chemistry and Leaf Litter Decomposition in Terrestrial Ecosystems: A Triangular Relationship. Oikos 79(3):439-449.

Bridson, J.N., 1985. Lipid fraction in forest litter: Early stages of decomposition. Soil Biology & Biochemistry 17(3): 285-290.

Carter, D.O., Yellowless, D., Tibbett, M., 2007. Cadaver Decomposition in Terrestrial Ecosystems. Naturwissenschaften 94:12–24.

Council directive: EWG guideline 90/496/EEC, 1990. Official Journal of the European Communities L 276:40-44.

Enser, M., Hallett, K., Hewett, B., Fursey, G.A.J., Wood, J.D., 1996. Fatty acid content and composition of English beef, lamb and pork at retail. Meat Science, 44, 443–458.

Gotaas, H.B., 1956. Composting: sanitary disposal and reclamation of organic wastes. Geneva: World Health Organization. 205 p.

Hendriks, W.H., Butts, C.A., Thomas, D.V., James, K.A.C., Morel, P.C.A., Verstegen, M.W.A., 2002. Nutritional Quality and Variation of Meat and Bone Meal. Asian Australasian Journal of Animal Sciences 15(10):1507-1516.

Khudzari, J.M., Tartakovsky, B., Raghavan, G.S.V., 2016. Effect of C/N ratio and salinity on power generation in compost microbial fuel cells. Waste Management 48: 135-142.

Longvah, T., Deosthale, Y.G., 1998. Compositional and nutritional studies on edible wild mushroom from Northeast India. Food Chemistry 63(3):331-334.

Mouginot, C., Kawamura, R., Matulich, K.L., Berlemont, R., Allison, S.D., Amend, A.S., Martiny, A.C., 2014. Elemental stoichiometry of Fungi and Bacteria strains from grassland leaf litter. Soil Biology and Biochemistry 76:278–285.

Raksakantong P., Meeso N., Kubola J., Siriamornpun S., 2010. Fatty acids and proximate composition of eight Thai edible terricolous insects. Food Research International 43:350–355.

Rawlins, A.J., Bull, I.D., Poirier, N., Ineson, P., Evershed, R.P., 2006. The biochemical transformation of oak (*Quercus robur*) leaf litter consumed by the pill millipede (*Glomeris marginata*). Soil Biology & Biochemistry 38, 1063-1076.

Sadler, M., 2003. Nutritional properties of edible fungi. Nutrition Bulletin 28(3):305 – 308.
